# Evaluation of a hybrid telehealth care pathway for patients with axial spondyloarthritis including self-sampling at home: results of a longitudinal proof-of-concept mixed-methods study (TeleSpactive)

**DOI:** 10.1007/s00296-024-05581-w

**Published:** 2024-04-11

**Authors:** Hannah Labinsky, Susann May, Katharina Boy, Sophie von Rohr, Manuel Grahammer, Sebastian Kuhn, Jessica Rojas-Restrepo, Ekaterina Vogt, Martin Heinze, Georg Schett, Felix Muehlensiepen, Johannes Knitza

**Affiliations:** 1 Department of Internal Medicine 3, Rheumatology and Immunology, Friedrich-Alexander University Erlangen-Nürnberg and Universitätsklinikum Erlangen, Erlangen, Germany; 2 Department of Internal Medicine 2, Rheumatology/Clinical Immunology, University Hospital Würzburg, Oberdürrbacher Straße 6, Würzburg, Germany; 3 Center for Health Services Research, Faculty of Health Sciences Brandenburg, Brandenburg Medical School Theodor Fontane, Rüdersdorf bei Berlin, Germany; 4 Abaton GmbH, Berlin, Germany; 5 Institute for Digital Medicine, University Hospital of Giessen and Marburg, Marburg, Germany; 6 Thermo Fisher Scientific, Freiburg, Germany; 7 Department of Psychiatry and Psychotherapy, Brandenburg Medical School, Immanuel Hospital Rüdersdorf, Rüdersdorf, Germany; 8 AGEIS, Université Grenoble Alpes, Grenoble, France

**Keywords:** Axial spondyloarthritis, axSpA, Electronic patient-reported outcome, ePRO, Treat-to-target, Shared decision-making, Surveys and questionnaires

## Abstract

Patients with axial spondyloarthritis (axSpA) require close monitoring to achieve the goal of sustained disease remission. Telehealth can facilitate continuous care while relieving scarce healthcare resources. In a mixed-methods proof-of-concept study, we investigated a hybrid telehealth care axSpA pathway in patients with stable disease over 6 months. Patients used a medical app to document disease activity (BASDAI and PtGA bi-weekly, flare questionnaire weekly). To enable a remote ASDAS-CRP (TELE-ASDAS-CRP), patients used a capillary self-sampling device at home. Monitoring results were discussed and a decision was reached via shared decision-making whether a pre-planned 3-month on-site appointment (T3) was necessary. Ten patients completed the study, and eight patients also completed additional telephone interviews. Questionnaire adherence was high; BASDAI (82.3%), flares (74.8%) and all patients successfully completed the TELE-ASDAS-CRP for the T3 evaluation. At T3, 9/10 patients were in remission or low disease activity and all patients declined the offer of an optional T3 on-site appointment. Patient acceptance of all study components was high with a net promoter score (NPS) of +50% (mean NPS 8.8 ± 1.5) for self-sampling, +70% (mean NPS 9.0 ± 1.6) for the electronic questionnaires and +90% for the T3 teleconsultation (mean NPS 9.7 ± 0.6). In interviews, patients reported benefits such as a better overview of their condition, ease of use of telehealth tools, greater autonomy, and, most importantly, travel time savings. To our knowledge, this is the first study to investigate a hybrid approach to follow-up axSpA patients including self-sampling. The positive results observed in this scalable proof-of-concept study warrant a larger confirmatory study.

**Supplementary Information:**

The online version contains supplementary material available at 10.1007/s00296-024-05581-w.

## Introduction

Axial spondyloarthritis (axSpA) is a common chronic inflammatory rheumatic disease primarily affecting the axial skeleton and potentially other joints and organs [1]. Regular monitoring of disease activity using validated composite scores such as the ASDAS-CRP under the supervision of a rheumatologist has been defined as a quality standard [2].

Developments such as the global shortage of medical specialists and the increasing prevalence of rheumatic patients make it difficult to apply quality standards in clinical practice. To compensate these shortages, care delivery needs to be designed more efficiently. Telehealth promises to realize the quadruple healthcare aim of enhancing patient and provider experience, improving population health, and reducing costs [3]. The Covid-19 pandemic has accelerated telehealth implementation in rheumatology [4] and led to the publication of official recommendations by the European league against rheumatism (EULAR). A supporting systematic review [5], however, revealed the small number of rheumatic telehealth studies available. A landmark study in patients with rheumatoid arthritis (RA) demonstrated that remote monitoring of patients enabled a safe reduction of face-to-face (F2F) visits and associated costs [6]. In this study, nurses or rheumatologists reviewed electronic patient-reported outcomes (ePROs) and C-reactive protein (CRP) results to decide whether a F2F visit was necessary. ePROs could be collected by the patient anywhere and anytime; however, patients still had to see a healthcare professional (HCP) to generate the necessary CRP result.

Despite the merits of remote care, it is essential to acknowledge the undeniable advantages of on-site visits. A hybrid care approach acknowledges the complementary nature of both modalities, harnessing the strengths of remote and in-person care. Notably, a randomized controlled study (RCT) [7] focusing on RA has demonstrated that a hybrid care model is non-inferior to traditional in-person consultations. However, the application of such a hybrid model to axSpA has not been explored yet and remote monitoring studies of axSpA are in general scarce [8–10]. Despite CRP being an integral part of the gold standard, ASDAS-CRP, remote care studies are limited to questionnaire-based ePROs and do not include CRP [8]. We previously demonstrated that at-home self-sampling was highly accepted by other rheumatic patient groups and resulted in accurate laboratory results [11–13]. To enable a home-based patient-derived ASDAS-CRP seems highly feasible, but has not yet been demonstrated. The aim of this proof-of-concept study (TeleSpactive) was to investigate (1) the feasibility of a hybrid telehealth care axSpA pathway including a remote ASDAS-CRP (TELE-ASDAS-CRP), based on upper-arm CRP self-sampling at home and (2) the patient perspective.

## Methods

### Study design

A mixed-methods proof-of-concept study assessing (1) the feasibility of the hybrid telehealth care axSpA pathway including TELE-ASDAS-CRP over 6 months and (2) the patient perspective based on qualitative patient interviews. The study was approved by the institutional review board (IRB) of the Medical Faculty of the University of Erlangen-Nürnberg, Germany (21-357-B) and conducted in compliance with the Declaration of Helsinki. All patients provided written informed consent prior to study participation.

### Patients

Consecutive patients from the outpatient clinics of the Department of Rheumatology at the University Hospital Erlangen were included if they fulfilled the following inclusion criteria: confirmed axSpA diagnoses, informed consent, stable disease without any therapy changes for at least 6 months, minimum age of 18 years, sufficient language skills, and regular usage of a smartphone. Exclusion criteria were an unstable disease and unwillingness or inability to comply with the protocol.

### Hybrid telehealth care axSpA pathway

**Fig. 1:**
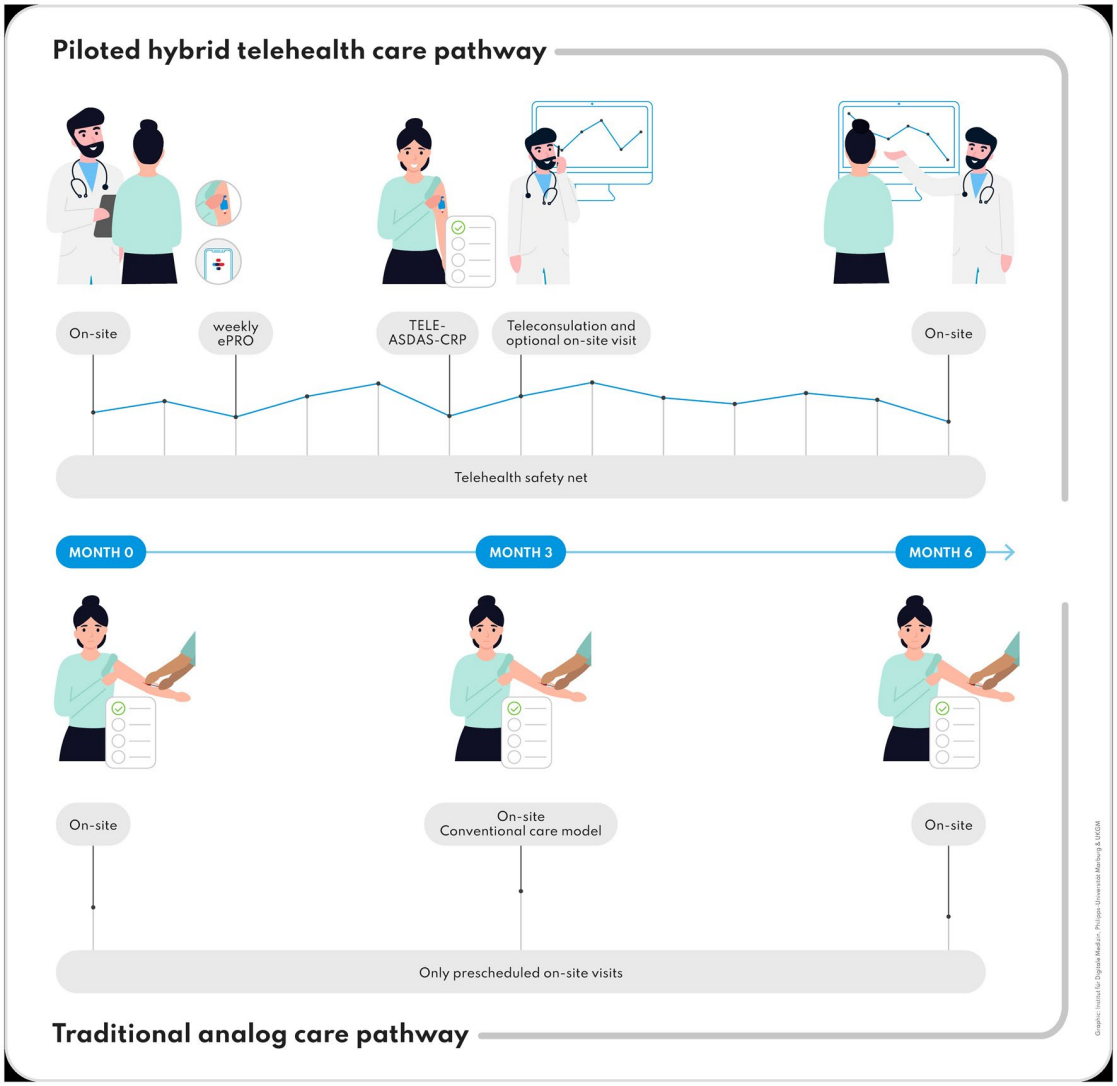
Piloted hybrid telehealth care pathway compared to traditional analogue care pathway. The figure was created with the Adobe illustrator software, version 2024 (28), Republic of Ireland

Figure 1 depicts an overview of the piloted pathway compared to traditional care. During a regular on-site visit (T0), a CE (conformité europenne)-certified medical smartphone application (ABATON) was installed on the patient’s smartphones. Patients used this app to complete questionnaires: a weekly two-question flare questionnaire (“Have you had a disease flare in the last week?” and “How many days did the flare last?”), a bi-weekly Bath Ankylosing Spondylitis Disease Activity Index (BASDAI), and a bi-weekly patient global disease activity (PtGA) score. If the patient forgot to answer the questionnaire, he was reminded to answer on 3 consecutive days. At any time, both the patient and the physician had access to the questionnaire results.

At T0, patients were instructed to use an upper-arm capillary self-sampling (TAPII, YourBio Health, Medford, USA). The self-sampling procedure has been described previously [13]. Briefly, patients apply the heat pack for 1 min, disinfect the area and attach the device to the upper arm. Pressing a button causes microneedles to puncture the skin and an applied vacuum to automatically collect capillary blood. The blood sealed in the collection tube was shipped to Thermo Fisher Scientific (Freiburg, Germany) for analysis. CRP analysis was done using the B·R·A·H·M·S CRPus KRYPTOR test kit (Thermo Fisher Scientific, Hennigsdorf, Germany). The CRP test results were provided by Thermo Fisher to the treating rheumatologist. At T0, patients received an extra upper-arm capillary self-sampling (TAPII, YourBio Health, Medford, USA), a heat pack, and a prepaid return parcel to enable a remote ASDAS-CRP (TELE-ASDAS-CRP) approximately 10 weeks after T0.

A telephone call after 3 months (T3) was pre-scheduled to discuss remote monitoring results and an additional on-site appointment was offered. At T6, 6 months after baseline, a pre-scheduled local appointment was held where the rheumatologists could also access the remote monitoring results.

### Outcomes

Disease activity was assessed using ASDAS-CRP at T0, T3, and T6. Monitoring adherence was assessed as percentage of completed questionnaires. Completion, punctuality, and analyzability of TELE-ASDAS-CRP was investigated. Uptake of offered T3 on-site visits was analyzed. Patient acceptance of the medical app, the self-sampling device, telephone consultation, and whole hybrid pathway was evaluated using the net promoter score (NPS) [14]. The NPS is based on an 11-point numeric rating scale (0–10). Answers between 0 and 6 are categorized as detractors, 7 and 8 as passives, and 9 and 10 as promoters. The NPS is equal to the percentage of promoters subtracting the percentage of detractors.

### Qualitative interviews

Qualitative interviews were conducted to explore patients’ experiences. To reduce patient burden and risk of infection, the interviews were conducted by telephone. The interviews took place between October and December 2022. The semi-structured interview guide (Supplementary Table 1) consisted of open-ended questions, with initial opening questions refined by follow-up questions. The interview guide was developed by health services researchers (SM, FM) and physicians (HL, JK) in an iterative review process. Prior to commencing interviews, the interview guide was tested and refined in three pilot interviews. No revisions were necessary. The final interview guide included the following topics: experiences with, acceptance of, benefits/drawbacks of and transferability to standard care of (A) the medical smartphone application (ABATON) and (B) capillary blood self-sampling (Supplementary Table 1). Data collection and analysis was conducted by a MD student (KB) and two healthcare researchers (SM and FM) based on Kuckartz’s structured qualitative content analysis [15]. After transcribing the audio material, the data were analyzed using MAXQDA software (Verbi GmbH). Representative quotes were selected from the transcripts, translated into English, and included in the manuscript. The precise interview analysis has been previously described [16, 17].

### Statistical analysis

No formal sample size calculation was performed due to the exploratory proof-of-concept character of the study and limited funding. To enable in-depth insights despite the limited sample size, patients were included to complete qualitative interviews. Statistical analysis was performed using Microsoft Excel 2019 and GraphPad Prism 8. *P* values less than 0.05 were considered significant. Patient-to-patient comparisons were summarized by median and range (minimum value to maximum value) for interval data and as absolute (*n*) and relative frequency (percent) for nominal data. Statistical differences were assessed by Mann–Whitney *U* test. Results were reported following the STAndards for the Reporting of Diagnostic accuracy studies guideline.

## Results

### Patient characteristics

All included patients (Table 1) had a history of axSpA: 2/10 patients also had a history of peripheral joint involvement, 5/10 patients were female. Median age was 38.5 (range: 27–64) years. Median disease duration was 4.5 (range: 1–15) years. Seven of ten patients received TNFi therapy (one patient in combination with MTX), two of ten patients NSAIDs, and one patient did not receive any therapy. At baseline, the disease activity was low (median BASDAI 1.0 (range: 0–3.6) and median ASDAS-CRP 1.4 (range: 1.0–2.1)).

**Table 1:** Patient characteristics

Pat	Sex	Age at enrollment (years)	Appr. disease duration (years)	HLA-B27	Therapy	Education*	Occupation*	Qualitative interview performed
1	M	52	12	Positive	TNFi	Unclear	Unclear	No
2	F	44	1	Positive	NSAIDs	Secondary school diploma	Practice manager	Yes
3	F	64	4	Positive	TNFi	Secondary school diploma	Pensioner	Yes
4	F	28	2	Positive	NSAIDs	Secondary school diploma	Kindergarten teacher	Yes
5	M	34	1	Positive	No	Secondary school diploma	Paramedic	Yes
6	M	27	15	Positive	TNFi	University degree	Project manager	Yes
7	F	27	2	Negative	TNFi	Unclear	Unclear	No
8	F	42	5	Negative	TNFi, MTX	High school degree	Restaurateur	Yes
9	M	38	8	Positive	TNFi	University degree	Project manager	Yes
10	M	39	8	Positive	TNFi	University degree	Physician	Yes

*Pat.* patient, *M* male, *F* female, *appr.* approximate

^*^ Data were collected as part of the qualitative interview

### Telemonitoring application

**Fig. 2:**
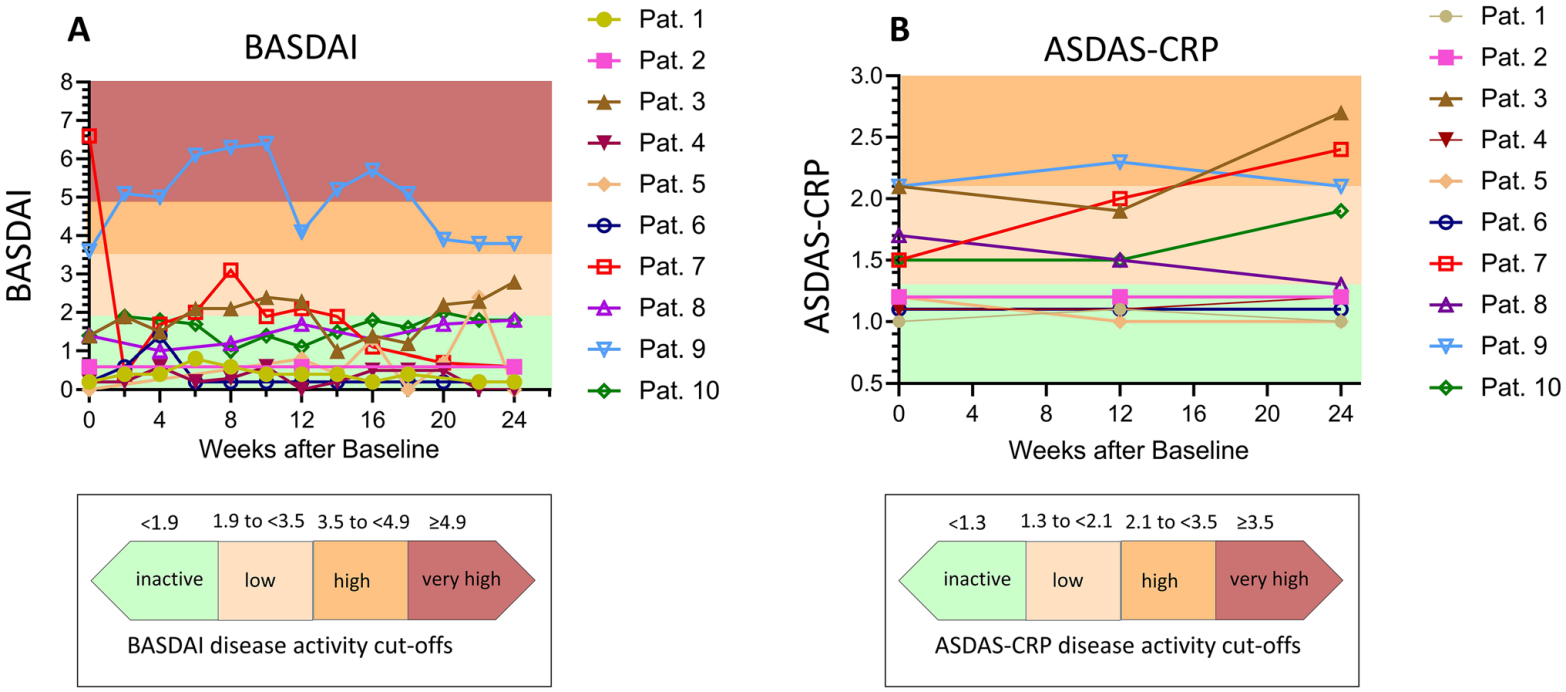
Remote monitoring results. BASDAI (**A**) and ASDAS-CRP (**B**) are presented. BASDAI was collected every 2 weeks over 6 months. ASDAS-CRP was collected at T0, T3, and T6. For the BASDAI [28] and the ASDAS-CRP, the level of disease activity is highlighted in color and explained below the graph in the legend, respectively. The color and symbolic representation of the ten patients is explained in the legend to the right of the graph. Missing data points show that the questionnaire was not completed at the respective time point

BASDAI and PtGA were documented every 14 days and the flare questionnaire every week between T0 and T6 using a CE-certified smartphone application (ABATON). Individual scores and trend graphs served were discussed at the telephone visit at T3 and at the on-site visit at T6. Adherence to electronic questionnaires was high with 82.3% electronic BASDAIs (107/130) and 74.8% (187/250) electronic flare questionnaires completed. Median pooled BASDAI (*n* = 107) was relatively low with 1.1 (range: 0–6.4). The BASDAI (Fig. 2A), ASDAS-CRP (Fig. 2B), and the PtGA (Supplementary Fig. 1) trend curves are shown. The disease activity scores undulated, but remained relatively stable overall (median ΔASDAS-CRP 0.2, range: 0–0.9; median ΔBASDAI 1.1, range: 0–2.7; median PtGA 2, range: 0–7).

According to the ASDAS-CRP, five patients remained inactive throughout T0–T6. Two patients had low disease activity at all three time points. Two patients already had high disease activity at T0 according to ASDAS-CRP and also at T6. One patient had low disease activity at T1 and T3 and high disease activity at T6 (Patient 7). However, none of the patients required a change in treatment even at T6.

In response to the question of whether a flare had occurred in the last week, there were a total of 10 yes answers and 240 no answers. During the study period of 6 months, four patients reported not having had a single flare in the last week, four patients reported one flare, one patient reported two flares, and one patient reported four flares. None of the reported flares lasted longer than 5 days. The number and mean length of the reported flares are shown in Supplementary Fig. 2.

### Self-sampling of capillary blood

The first capillary blood sample was taken during the on-site appointment in the study outpatient clinic and was performed by the patients with assistance. Venous blood sampling revealed a negative CRP (<5.1 mg/l) in all study patients at baseline. Capillary blood self-sampling was successful in 9/10 patients at baseline. In one patient, the capillary blood was not sufficient for laboratory analysis. The capillary CRP determination correlated perfectly (100% concordance) with the venous (negative CRP in capillary and venous laboratory analysis in 9/9 tests).

The second capillary blood test was performed by the patient at home and without assistance. All ten study patients managed blood self-sampling, and CRP determination was successful from all capillary blood samples after mailing to the analysis laboratory. However, one patient did not succeed in self-sampling with the first device handed out and only succeeded after a second self-sampling device was supplied (according to the patient due to a defective device).

The time of second self-sampling was agreed with the patients to be 2 weeks before the telephone visit. Setting of an appointment reminder on the patient’s own smartphone was suggested. Seven of ten patients carried out the self-sampling and mailing of their samples independently. After three patients had not yet submitted a sample to the analysis laboratory 1 week before the telephone visit, these patients were reminded by phone.

### Teleconsultation

The teleconsultation was carried out approx. 3 months (T3) after the first on-site appointment. The appointment had been arranged in advance with the patient and all except for one patient were available at the agreed time. The teleconsultation was rescheduled with the patient who was unable to attend. During the telephone visit, the current condition and the telemonitoring results (BASDAIs, relapse questionnaires, ASDAS-CRP) were discussed. According to the ASDAS-CRP, 9/10 were in remission or showed low disease activity at T3. A regular on-site appointment was offered to all patients at T3, but was not requested by any of the patients.

### Patient acceptance

Patient acceptance of all study components was high with NPS of +50% (mean NPS 8.8 ± 1.5) for capillary self-sampling, +70% (mean NPS 9.0 ± 1.6) for the ePRO smartphone application, and +90% for the teleconsultation (mean NPS 9.7 ± 0.6).

### Qualitative interviews

The qualitative interviews investigated the feasibility of telemonitoring for patients with stable axSpA, focusing specifically on their experiences with this technology.

For the research, ten interviews were initially planned. However, only eight were conducted as two patients, despite having scheduled appointments, could not be reached after multiple attempts and consequently had to be excluded from the study. Median age of interviewed patients was 38.5 (range: 27–64) years, see Table 1. The genders of the patients were equally represented (four female/four male). The patients reported different professional and educational backgrounds. All patients had a diagnosis of axSpA. The interviews lasted between 10 and 18 min.

### Current challenges with the traditional patient pathway in contrast to the telemonitoring assisted pathway

Long waiting times at the clinic, as well as long traveling distances were described as inconvenient and challenging in the context of traditional healthcare. Patients reported noticeable changes regarding their rheumatology care through telemedicine.

I used to have to go in maybe every 3 or 4 months, to have my blood taken and so on, so that they could keep track of how my relapse was going and whether I had one. It just makes it easier for me because I drive 45 minutes to the clinic. That’s just a bit better for me in terms of time. And I also have a bit more contact, let’s say, with the doctor. Because if there’s anything more serious, she gets in touch with me without me having to go to the clinic. That makes things a bit easier for me, of course. (P 3, pos. 14–15)

### Experiences with the feasibility of telemonitoring with stable axSpA

Patients positively highlight the ease of use associated with the smartphone application telemonitoring. They also appreciate receiving a comprehensive overview of their symptom progression, which they find helpful.

This reflects it more realistically, you can definitely say that I myself also have the opportunity to follow a course, to see how I have answered and to see my own course is also an exciting point, which also helps me to assess and classify the whole thing a little better. (P7, pos. 43)

In addition, the ability to complete formerly paper questionnaires digitally is highlighted as a significant positive development.

Well, they’re exactly the same questionnaires that I always fill out by hand in the doctor’s clinic, so I think it’s great that it’s somehow recorded digitally and I’m not ticking five pages every time and then wondering whether anyone will ever look at it. Yes, and so, I think, it will certainly go into the system somewhere and it will also be visualized in this/yes, in a graphic like this. (P 8, Pos. 11)

Patients suggest that the data entered should be accessible to all healthcare professionals providing treatment to ensure uninterrupted treatment.

But I think the app’s approach is great, especially with the background that my experiences and parameters are entered directly. One point, which is of course also due to the early phase of the app, is that even in the hospital there is often confusion as to who actually has the data and all colleagues can really see it, so if you’re not just being treated by a rheumatologist, but on a ward, as is the case with me. (P 7, Pos. 37)

### Experiences with the feasibility of capillary self-sampling with axSpA

Patients described the independent collection of capillary blood as user friendly. The instructions were considered as clear, especially supported by the illustrations attached.

Patients suggested that more information on the transport of the sample and laboratory analysis would be helpful.

I took the blood in the spring. I don’t know what will happen to the blood in the warm season. Will it go bad or break, how long it will be in transit. I did send it straight to the post office, but when does it arrive at the lab? I’m not an expert, I don’t know, does the blood go bad or does it get warm? Transparency with regard to the transportation route and the transportation time- that’s what concerns me, whether everything is correct. (P 2, pos. 63–68)

It was also described as helpful that the first blood sample is taken in the presence of a HCP to avoid sources of error.

Ecological aspects were also critically addressed. According to the patients, capillary self-sampling generates a lot of packaging waste and single-use products are utilized, in which patients see potential for further improvement.

### Future feasibility of the explored care pathway

Patients reported that they could imagine the explored pathway in their regular axSpA care for the future. In this context, interview participants repeatedly emphasized the benefits of the time and travel savings as particularly positive aspects.

Yes, of course. As I said earlier, why should I go there? We can all save us time and money and if there really is an abnormality or you have to make some kind of therapy adjustments or otherwise order medication, then you can find your way there. You have to, of course. But that’s exactly where I see the potential, yes. (P 6, pos. 58–59)

However, this would also require an adaption of structural processes in German health system. In this regard, the required referral to specialists and the presentation of the insurance card were mentioned as examples.

I think regular consultations with a doctor are important, in fact I don’t think they necessarily have to happen on site. So, a self-test plus a remote consultation with a doctor, where you can review the data together and discuss it, I could actually imagine that working well. That would also make it easier for me overall. Nevertheless, it’s currently a structural problem, a billing problem. In order to get my medication, I have to regularly present a referral form or my insurance card to the rheumatology department, for example. (P 7, pos. 59)

## Discussion

To the best of our knowledge, we are the first to present results of a hybrid axSpA care model, incorporating a remote and entirely patient-derived ASDAS-CRP.

While limited studies have explored rheumatic hybrid care models, a randomized controlled study for RA has already demonstrated that a hybrid care model is non-inferior to traditional in-person consultations [7]. In addition, the Digireuma study by Benavent et al. [10] already showcased the feasibility of a hybrid care model for RA and axSpA patients, utilizing a digital solution to collect patient-reported outcomes (PROs). Nevertheless, this study lacked independently conducted capillary blood sampling and qualitative evaluation.

Results of other randomized controlled hybrid care model studies are still pending. The ongoing large randomized controlled trial TeleSpA [18] is solely based on questionnaires. The ongoing Remonit study [19] included point-of-care (POC) CRP tests for a subgroup of 12 patients additional to questionnaire-based ePROs. This POC device, QuikRead go [20], provides exact and fast CRP results, however was not designed for patient use and high costs prevent implementation in routine care. Semiquantitative POC devices, such as those used in the TELERA study [21], are more affordable, however only indicate ranges of CRP results (i.e., 5–10 mg/l). All of these CRP tests are also based on conventional finger-pricking. We could previously demonstrate that affordable upper-arm self-sampling devices cause significantly less pain, were preferred by patients and produced accurate CRP results [13]. Our results were consistent with this.

Recent research affirm the equivalence and preference of ePRO over paper-based methods in rheumatology patients [22]. In our study, ePROs were collected at weekly and bi-weekly intervals. The strong adherence to ePROs over 6 months aligns with previous results [23]. The high acceptance of ePROs in our patient cohort with a NPS exceeding 70% underscores the success of our approach.

Rather low undulations of the activity scores did not require any therapeutic adjustment or additional on-site appointments. No patient requested an additional on-site visit or therapeutic changes during the teleconsultation at T3. The shared interpretation of the disease activity scores by patients and physicians in the telephone interview is a strength of our hybrid care model. In addition to the validated BASDAI and ASDAS-CRP scores, we used a self-administered flare questionnaire consisting of two questions, which demonstrated a high degree of absence of flares in our study patients. However, the relapse questionnaire still needs to be validated in a larger cohort of SpA patients.

For rheumatologists, the pathway enables continuous granular patient monitoring including objective CRP values to provide better informed and timely treatment decisions. Importantly this pathway would enable need-adapted and even patient initiated follow-up (PIFU) visits, setting free capacities to see patient with higher disease activities.

Remarkably, our study demonstrates exceptionally high patient acceptance of self-sampling, electronic questionnaires, and teleconsultation, surpassing even our previous studies [9, 11, 24]. Telephone interviews, employing a mixed-methods approach, delved into the factors that underpin this acceptance. They highlighted the significance of time and cost savings, alongside the promotion of disease awareness through the increased frequency of measurements and the presentation of trend curves directly to the patients.

Despite these successes, our study has limitations. The small patient cohort impacts the quantitative data’s generalizability but allows for a comprehensive mixed-methods approach. In addition, the relatively young patient cohort raises concerns about digital literacy and smartphone possession as potential barriers. The study focused on clinically stable patients over a 6-month period, and longer, larger studies are crucial for further validation and exploration. The lack of physical examination is a major limitation of this pathway. Including digital biomarkers such as the dorsal finger fold index [25] to detect arthritis, stepcount [26] to predict flares, and sensor-based spine mobility measurement [27] could enhance this pathway. A health economic evaluation is crucial to demonstrate actual cost savings of this pathway. It is essential to qualitatively examine healthcare professionals’ perceptions of the new pathway to develop one that is both sustainable and widely accepted.

## Conclusion

Our results demonstrate the feasibility, high patient acceptance, and overall potential of a scalable axSpA hybrid telehealth care pathway, incorporating an entirely remote ASDAS-CRP evaluation. This innovative pathway could relief overburdened inflexible rheumatology care systems by reducing the strain of unnecessary in-person consultations for both patients and the healthcare system. Furthermore, the pathway enables treat-to-target remote monitoring and empowers patients to perform a need-adapted ASDAS-CRP evaluation at home. This pathway offers a blueprint for a hybrid monitoring pathway transferrable to other rheumatic disease and beyond. Larger studies are needed to confirm the promising results.

### Supplementary Information

Below is the link to the electronic supplementary material.

Supplementary figure 1. Remote PtGA trend. The PtGA trend is presented. PtGA was collected every 2 weeks over 6 months. The colour and symbolic representation of the 10 patients is explained in the legend to the right. Missing data points show that the questionnaire was not completed at the respective time point. (JPG 1170 KB)

Supplementary figure 2. Number of flares and flare duration. The number of indicated flares out of a maximum of 25 possible flares is shown individually for each patient (2A). The mean duration of all indicated flares is shown in 2B. (PDF 596 KB)

Supplementary table 1. Patient interview guide (JPG 1010 KB)

## Data Availability

All data relevant to the study are included in the article or uploaded as supplementary material. Data analyzed during the current study are available from the corresponding author on reasonable request.

## Declarations

### Previous related congress abstract publication

The authors declare that there are no previous related congress publications.
