# Supplementary figures and images for: Evaluation of a hybrid telehealth care pathway for patients with axial spondyloarthritis including self-sampling at home: results of a longitudinal proof-of-concept mixed-methods study (TeleSpactive)
Source: Rheumatol Int. 2024 Apr 11;44(6):1133–42. doi: 10.1007/s00296-024-05581-w (PMC11108867; doi:10.1007/s00296-024-05581-w)

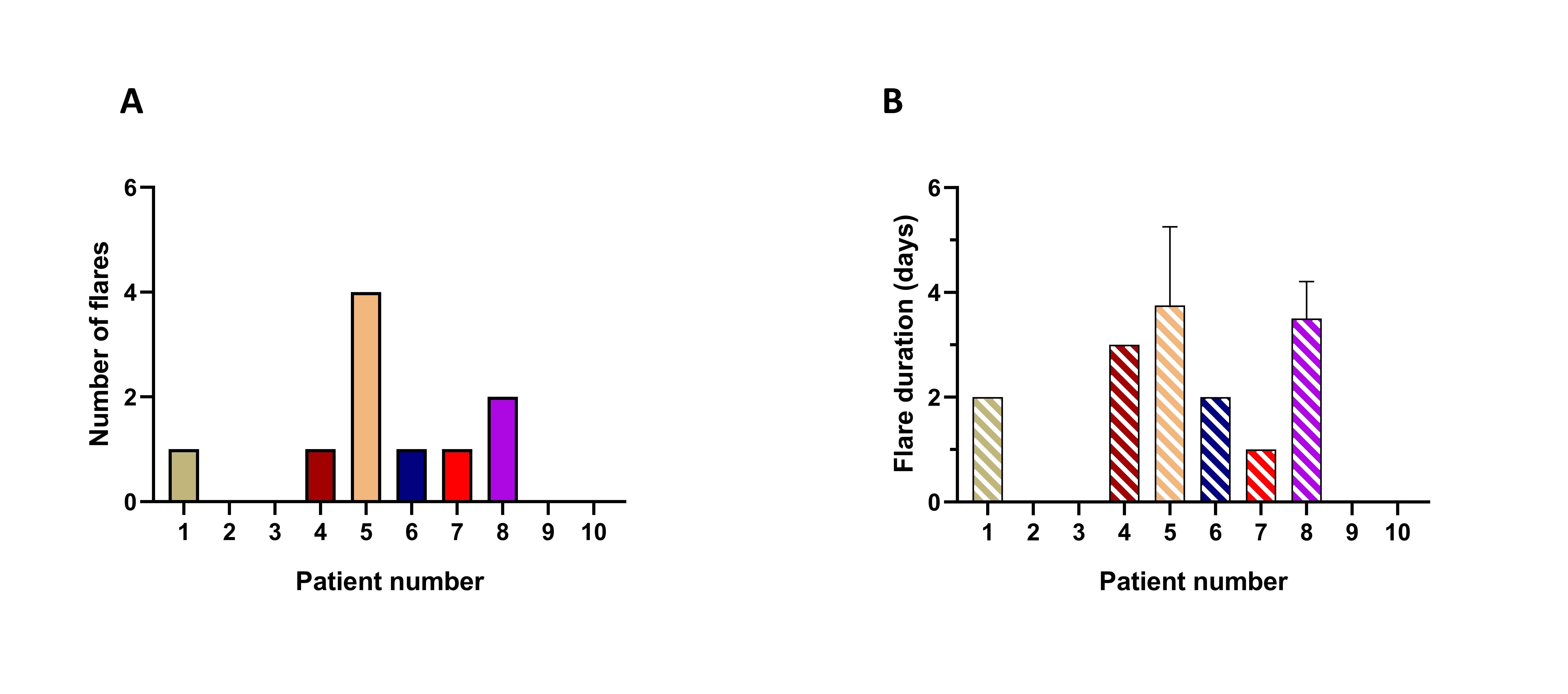

Supplement: Supplementary file 1 — Supplementary figure 1. Remote PtGA trend. The PtGA trend is presented. PtGA was collected every 2 weeks over 6 months. The colour and symbolic representation of the 10 patients is explained in the legend to the right. Missing data points show that the questionnaire was not completed at the respective time point. (JPG 1170 KB) [file 296_2024_5581_MOESM1_ESM.jpg]

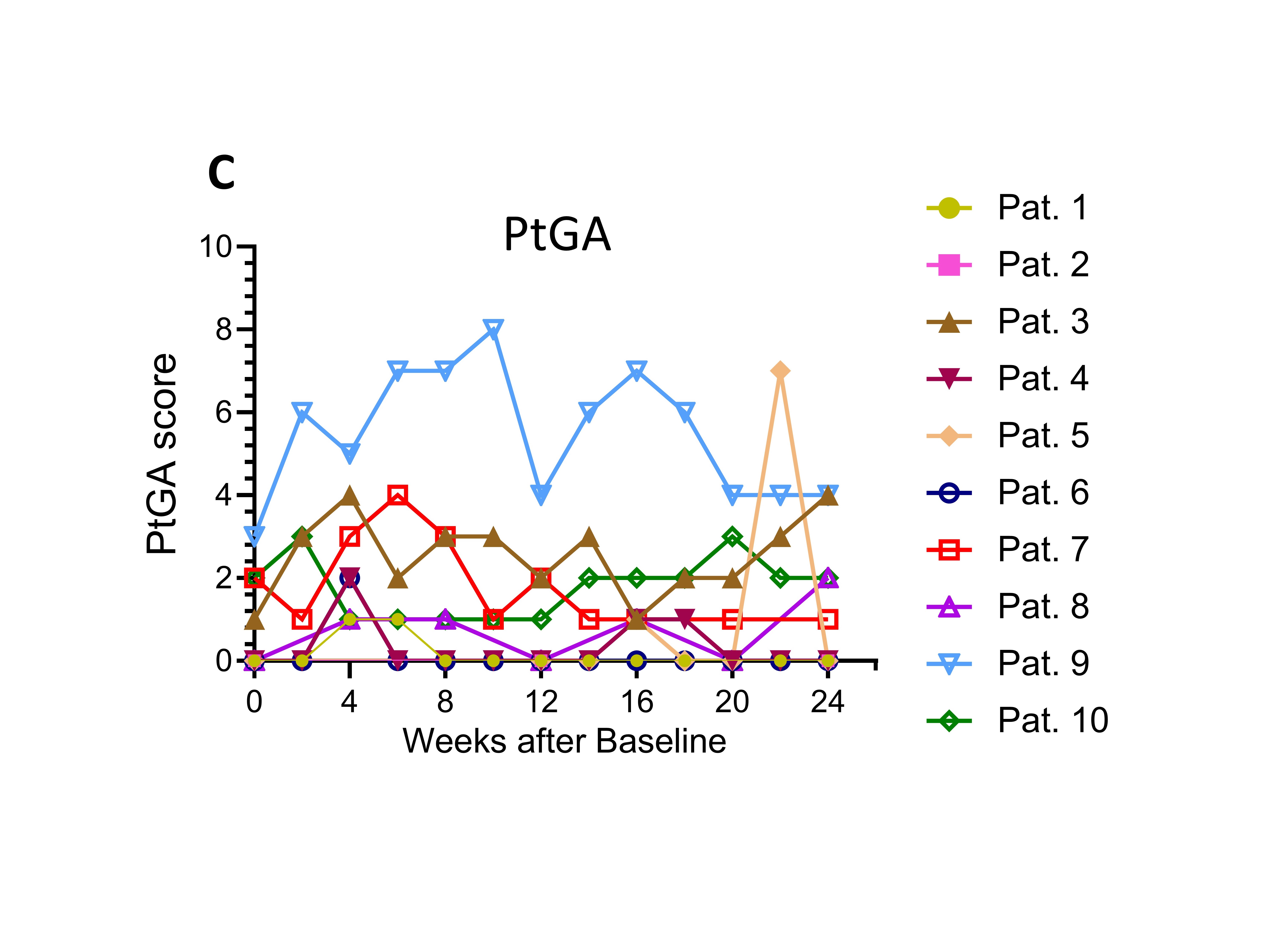

Supplement: Supplementary file 3 — Supplementary table 1. Patient interview guide (JPG 1010 KB) [file 296_2024_5581_MOESM3_ESM.jpg]
