# Supplementary material for: Evaluation of a hybrid telehealth care pathway for patients with axial spondyloarthritis including self-sampling at home: results of a longitudinal proof-of-concept mixed-methods study (TeleSpactive)
Source: Rheumatol Int. 2024 Apr 11;44(6):1133–42. doi: 10.1007/s00296-024-05581-w (PMC11108867; doi:10.1007/s00296-024-05581-w)
Supplement: Supplementary file 2 — Supplementary figure 2. Number of flares and flare duration. The number of indicated flares out of a maximum of 25 possible flares is shown individually for each patient (2A). The mean duration of all indicated flares is shown in 2B. (PDF 596 KB) [file 296_2024_5581_MOESM2_ESM.pdf]

Supplementary table 1. Patient interview guide

<table>
<tr>
<td>You have taken part in the following study:
"TELESpActive - A TELEmedicine study for screening and medical care in axial spondyloarthritis"

Please describe what you did in the study.</td>
<td rowspan="4">Can you tell us more about this?

And then?
How was that for you?

How do you see that? Can you please go into this in more detail?

Could you please give an example?

What do you mean in concrete terms?

Can you tell us more about this?

And then?
How was that for you?

How do you see that? Can you please go into this in more detail?

Could you please give an example?

What do you mean in concrete terms?</td>
</tr>
<tr>
<td>A Could you please describe your experience with the ABATON app?
× What works well? What doesn't?
× How does the app change your rheumatological care?
× Could you imagine continuing to use the app?
If yes, under what conditions? If not, why not?</td>
</tr>
<tr>
<td>B Could you please describe your experience with capillary blood sampling?
× If the blood test was commercially available, would you use it?
(to find out about your genetic risk (HLA-B27) and the necessary inflammation values)?
× Would you like to discuss the results with a doctor for categorisation?
× What advantages do you see in patients being able to take blood samples themselves at home? (if no suggestions are made e.g. shortening diagnosis, saving appointments etc...)
× What disadvantages do you see?</td>
</tr>
<tr>
<td>× Do you think digital monitoring of the disease with ABATON and blood sampling could usefully supplement on-site appointments?
× Do you think the continuous use of ABATON and blood sampling could save time during the actual on-site appointments?
× Do you think that the applications could also be helpful for other diseases?</td>
</tr>
</table>

× Could you imagine that your rheumatological care would only take place in this way?<br>➔ This means that you only go to a rheumatologist if necessary or if there are any abnormalities. Under what conditions?<br>o What are the advantages of rheumatism care as it is provided in the study?<br>o What disadvantages do you experience?	
× Is there anything else you would like to address?<br>× Have you noticed anything else?	

**Age**	**Gender**	**Since when SpA diagnosis?**
**Profession**	**Highest educational qualification**	**Notes**
